# Postoperative Pyoderma Gangrenosum Following Varicose Vein Surgery: Recognizing a Rare Surgical Mimic Before Extensive Tissue Loss

**DOI:** 10.7759/cureus.113733

**Published:** 2026-07-31

**Authors:** Omar El Salawi, Marc Dubois, Anthony De Smet

**Affiliations:** 1 Surgery, Antwerp University Hospital, Antwerp, BEL; 2 Vascular Surgery, Heilig Hart Ziekenhuis Lier, Lier, BEL

**Keywords:** diagnostic delay, pathergy, postoperative pyoderma gangrenosum, pyoderma gangrenosum, surgical site infection, varicose vein surgery

## Abstract

Postoperative pyoderma gangrenosum (PG) is a rare neutrophilic dermatosis that frequently masquerades as a surgical site infection. Because its clinical presentation closely resembles postoperative cellulitis or even necrotizing soft tissue infection, diagnosis is often delayed. Misdiagnosis may result in unnecessary antibiotic escalation, repeated surgical interventions, and progressive tissue destruction due to pathergy. This case highlights the importance of early recognition of PG and serves as a reminder for surgeons to consider this uncommon diagnosis when postoperative wounds fail to respond to conventional treatment.

A 75-year-old woman underwent elective open varicose vein surgery (ligation of neocrosse veins and phlebectomies) for recurrent symptomatic varicose veins of the left lower limb. One week postoperatively, she developed a painful erythematous wound that was initially diagnosed as a surgical site infection. Despite drainage, repeated wound care, and escalation from oral to broad-spectrum intravenous antibiotic therapy, the lesion rapidly progressed into an extensive necrotic ulcer. Repeated wound cultures and blood cultures remained sterile, while computed tomography scans demonstrated inflammatory changes without evidence of a drainable collection or necrotizing soft tissue infection. Following dermatological consultation, postoperative PG was suspected and confirmed by skin biopsy. High-dose systemic corticosteroid therapy was initiated, resulting in rapid clinical improvement, cessation of ulcer progression, and subsequent wound healing.

This case illustrates the diagnostic challenge of postoperative PG and emphasizes several clinical features that should prompt reconsideration of a presumed surgical site infection, including disproportionate pain, rapidly progressive ulceration, sterile cultures, failure of appropriate antimicrobial therapy, and inconclusive imaging findings. Early recognition is essential, as surgical manipulation may exacerbate disease progression through pathergy.

Postoperative PG should be considered in any patient presenting with a rapidly progressive postoperative wound that fails to improve despite appropriate antimicrobial treatment. Increased awareness among surgeons may facilitate earlier diagnosis, prevent unnecessary surgical procedures, and reduce the risk of extensive tissue loss.

## Introduction

The classical signs of inflammation-rubor, calor, dolor, and tumor-occurring in a postoperative wound are generally considered indicative of a surgical site infection. Surgical site infection is one of the most common postoperative complications encountered by surgeons. In the vast majority of cases, the diagnosis is straightforward and treatment with antibiotics and, when necessary, surgical drainage or debridement leads to clinical improvement. Rarely, however, a non-infectious inflammatory disorder may present with an almost identical clinical picture. Failure to recognize these conditions may result in inappropriate treatment and progressive tissue destruction [1].

Pyoderma gangrenosum (PG) is a rare neutrophilic dermatosis characterized by rapid progressive, painful, sterile ulceration of the skin. In the postoperative setting, PG frequently masquerades as a wound infection or even necrotizing soft-tissue infection [2,3]. Diagnosis remains challenging because no single diagnostic test exists; instead, PG is diagnosed using a combination of clinicopathological findings. Current consensus criteria require a biopsy demonstrating a sterile neutrophilic infiltrate as the major criterion, together with at least four minor criteria, including exclusion of infection, evidence of pathergy, characteristic ulcer morphology, rapid progression, associated systemic inflammatory disease, and a favorable response to immunosuppressive therapy. Because surgical trauma can exacerbate the disease through the phenomenon of pathergy, repeated wound exploration and debridement may accelerate tissue loss rather than improve healing [4].

We report on a patient who developed extensive postoperative ulceration following elective varicose vein surgery before the correct diagnosis of postoperative PG was established. The aim of this report is not only to describe a rare complication but also to remind surgeons that failure to recognize PPG early can have devastating consequences. Awareness of this uncommon diagnosis is essential to avoid unnecessary surgical interventions, initiate prompt immunosuppressive therapy, and prevent progression to extensive tissue destruction.

## Case presentation

A 75-year-old woman underwent elective open surgery for recurrent symptomatic varicose veins of the left lower limb. Her medical history included multiple previous varicose vein procedures and breast-conserving surgery with sentinel lymph node biopsy for left-sided breast carcinoma six years earlier, for which she remained on adjuvant tamoxifen therapy. Her regular medication included perindopril.

The procedure consisted of a crossectomy with ligation of the neocrosse, ligation of incompetent perforating veins, and staged ambulatory phlebectomies according to the Müller technique. The operation was uncomplicated, and the patient was discharged according to the standard postoperative protocol.

At routine outpatient follow-up seven days after surgery, she presented with increasing pain at one of the phlebectomy incisions. Clinical examination demonstrated a localized area of erythema, induration, and tenderness. Upon removal of the dressing, purulent-appearing fluid drained spontaneously from the wound. The clinical impression was that of an infected liquefied hematoma or postoperative surgical site infection. A wound swab was obtained for microbiological analysis, the wound was drained and irrigated with saline and povidone-iodine, and daily wound care with povidone-iodine packing was initiated. Empirical oral amoxicillin/clavulanic acid (875/125 mg three times daily) was prescribed.

Despite these measures, the patient's condition deteriorated rapidly. Two days later, she presented to the emergency department with severe progressive pain involving the left thigh and fever up to 38.5°C. Physical examination demonstrated increasing erythema, swelling, and induration surrounding the previously drained wound. She remained hemodynamically stable, with an oxygen saturation of 99%, blood pressure of 138/68 mmHg, normal peripheral perfusion, and a respiratory rate of 12 breaths per minute.

Laboratory investigations demonstrated leukocytosis (16.8 × 10³/µL) and an elevated C-reactive protein concentration of 74 mg/L. Repeat wound cultures, blood cultures, and routine laboratory investigations were obtained. Computed tomography (CT) of the left lower limb demonstrated diffuse skin thickening and inflammatory changes of the subcutaneous tissues without evidence of a drainable abscess, soft tissue gas, or necrotizing soft tissue infection (Figure 1).

**Figure 1:**
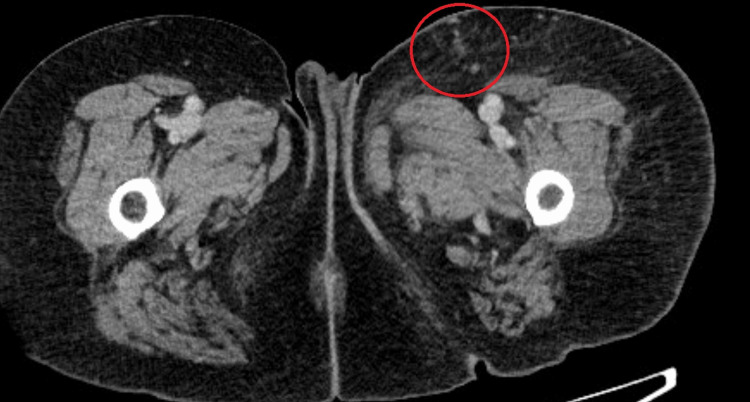
CT of lower limbs showing skin and subcutaneous inflammation without deeper inflammation or abscesses.

Given the presumed diagnosis of progressive postoperative cellulitis that had failed oral antibiotic therapy, the patient was admitted, and treatment was escalated to intravenous amoxicillin/clavulanic acid combined with clindamycin. Daily wound irrigation and local wound care were continued.

During the following 48 hours, however, the clinical picture became increasingly atypical. The wound enlarged rapidly, developing extensive superficial necrosis with relatively limited surrounding erythema but severe pain both at rest and on minimal palpation. Laboratory parameters deteriorated further, with the leukocyte count increasing to 25.9 × 10³/µL and the C-reactive protein concentration rising to 158 mg/L. Repeat wound cultures and blood cultures remained sterile. In consultation with the microbiology department, antimicrobial therapy was empirically escalated to intravenous piperacillin/tazobactam (Figures 2-4).

**Figure 2:**
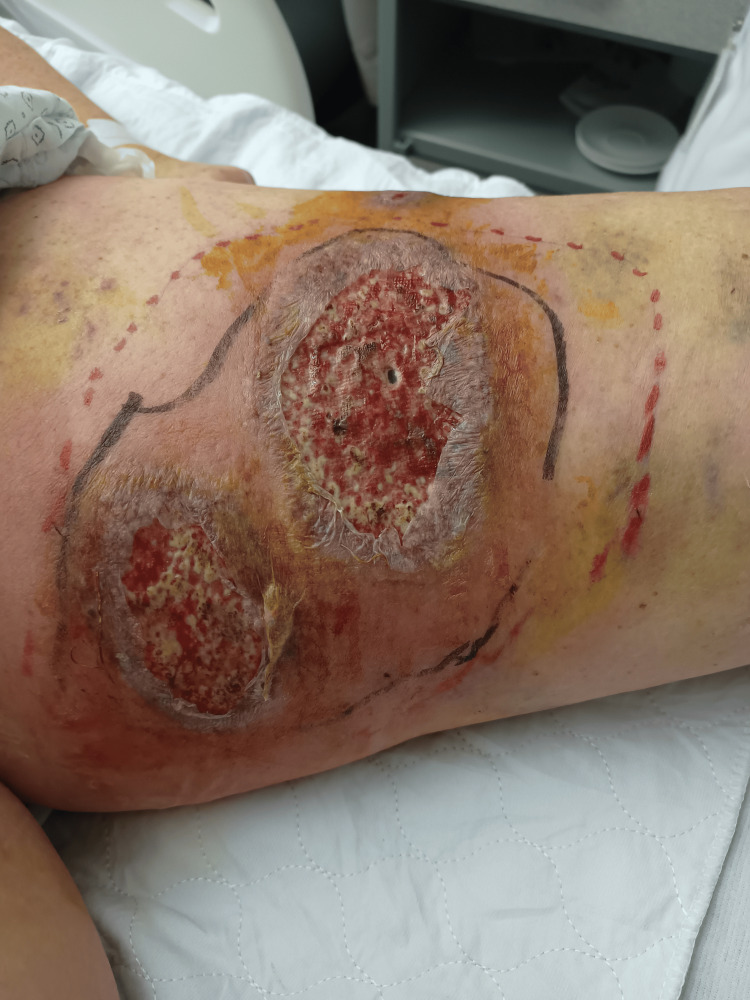
Admission day 2: ulcer progression after drainage of hematoma.

**Figure 3:**
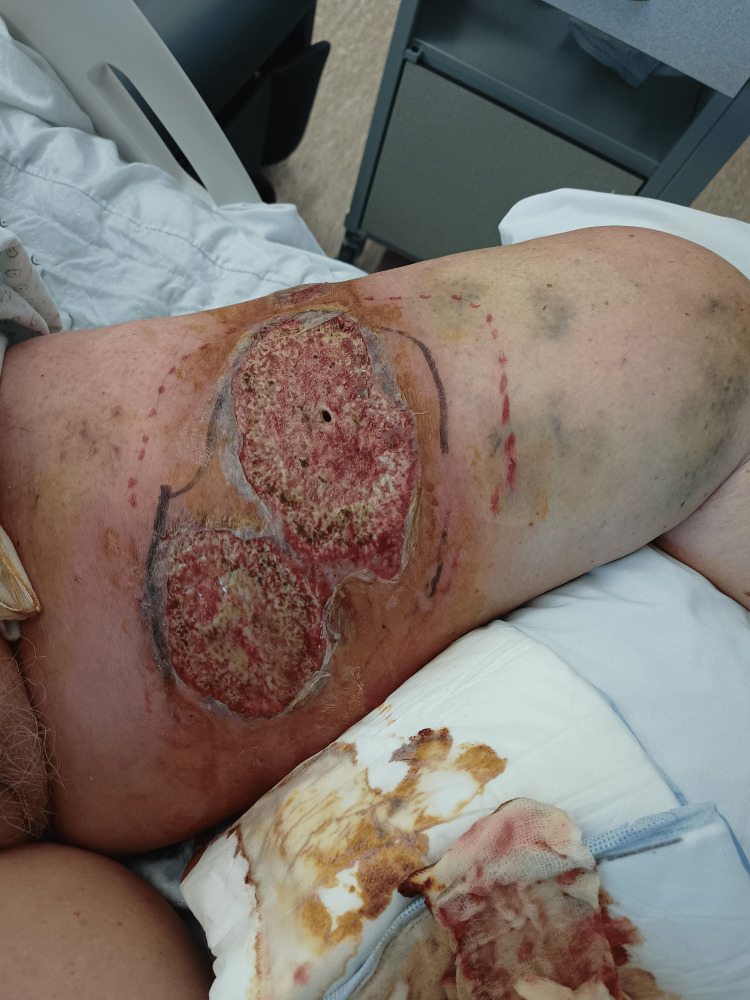
Admission day 3: rapid progression of necrosis and size of ulcer under expanded antibiotic treatment.

**Figure 4:**
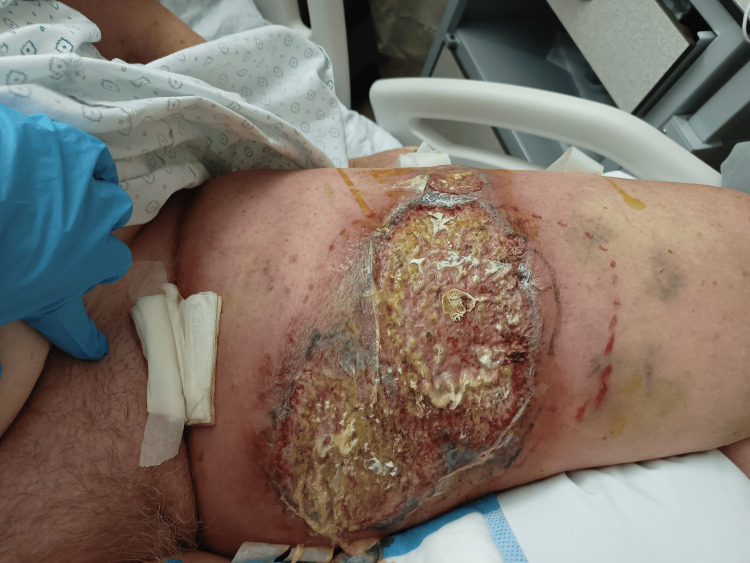
Admission day 4: continued rapid progression of necrosis and size of ulcer.

At this stage, several findings were no longer consistent with a conventional postoperative wound infection. Despite appropriate broad-spectrum antimicrobial therapy, the patient continued to deteriorate clinically and biochemically. Furthermore, repeated microbiological cultures remained sterile, while CT imaging failed to demonstrate a drainable collection. Surgical debridement was considered because of the rapidly progressive tissue necrosis but was ultimately deferred because the overall clinical presentation appeared atypical.

Over the subsequent days, the ulcer continued to expand, and severe pain persisted despite ongoing treatment. A repeat CT scan again demonstrated extensive inflammatory changes involving the skin and subcutaneous tissues, with only limited edema of the underlying musculature and no evidence of abscess formation or necrotizing fasciitis. Because of the combination of rapidly progressive ulceration, disproportionate pain, failure of appropriate antimicrobial therapy, repeatedly sterile cultures, and inconclusive imaging findings, the initial diagnosis of postoperative wound infection was reconsidered.

Given the concern for an atypical necrotizing soft tissue infection, consultation with a tertiary referral center was sought. However, transfer was not possible because of limited bed availability during the third wave of the COVID-19 pandemic in Belgium. Following multidisciplinary discussion involving infectious disease specialists and dermatologists, postoperative pyoderma gangrenosum (PPG) became the leading diagnostic consideration.

A skin biopsy was obtained from the wound edge, and the dermatology team initiated pulse corticosteroid therapy with intravenous methylprednisolone 1 g daily for three consecutive days because of the rapidly progressive clinical course and the strong clinical suspicion of PPG. This was followed by a tapering regimen of oral prednisolone starting at 64 mg daily, with dose reductions every two weeks depending on clinical response and wound evolution. At the same time, wound management was fundamentally changed. Gentle, non-traumatic wound care using absorbent foam and alginate dressings replaced conventional surgical wound management, and further debridement was deliberately avoided because of the risk of pathergy, a characteristic feature of PG in which even minor mechanical trauma may provoke an exaggerated inflammatory response, leading to rapid enlargement of existing ulcers or the development of new lesions. The temporal evolution of inflammatory markers is summarized in Table 1. Leukocyte count and C-reactive protein continued to rise during the first 48 hours after initiation of pulse corticosteroid therapy before declining rapidly, paralleling the patient's clinical improvement and arrest of ulcer progression (Table 1). 

**Table 1:** Evolution of inflammatory markers during hospitalization and timing of systemic corticosteroid therapy.

Hospital day	Leukocytes (×10³/µL)	CRP (mg/L)	Clinical remarks
1	16.8	74.0	Admission to the emergency department
2	25.9	158.2	-
3	26.3	211.0	-
4	32.0	198.0	Intravenous methylprednisolone pulse therapy initiated (1 g/day for three consecutive days; first dose administered in the evening)
5	39.6	256.0	Day 2 of intravenous methylprednisolone pulse therapy
6	55.3	262.0	Day 3 of intravenous methylprednisolone pulse therapy
7	37.1	104.0	Switched to oral prednisolone 64 mg/day (start of tapering regimen)
8	25.7	59.7	-
10	17.5	49.4	-
11	13.3	34.4	-
12	11.0	21.8	-
20	8.4	5.0	Discharged on tapering oral prednisolone regimen

Histopathological examination of the skin biopsy demonstrated a dense neutrophilic inflammatory infiltrate compatible with PG and showed no evidence of an infectious process or other alternative pathology (Figures 5-6). Although these findings are not pathognomonic, they strongly supported the clinical diagnosis of postoperative PG in conjunction with the patient's characteristic clinical presentation, repeatedly sterile microbiological cultures, and exclusion of other causes.

**Figure 5:**
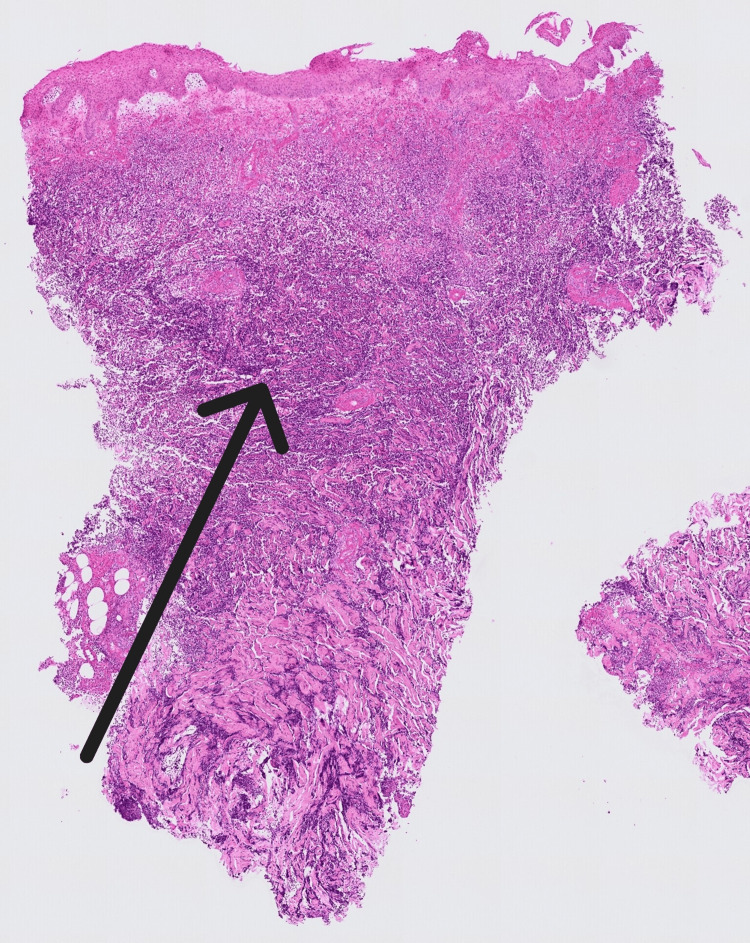
Pathology: H&E stain with 4× magnification showing a skin biopsy with hypercellular inflammation of the edge of the ulcer (many leukocytes marked with the arrow/purple coloring).

**Figure 6:**
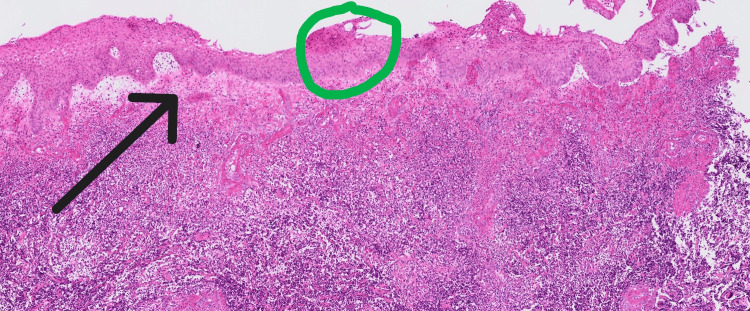
Pathology: H&E stain with 4× magnification showing subepidermal blister formation (arrow) and Superficial dermal edema and necrosis (circle).

Shortly after initiation of systemic corticosteroid therapy, further progression of the ulcer ceased. The patient's pain improved markedly, inflammatory markers gradually declined, and the wound progressively developed healthy granulation tissue. Repeated wound and blood cultures remained sterile throughout the admission, allowing discontinuation of antibiotic therapy after ten days of intravenous piperacillin/tazobactam.

Because of the high-dose corticosteroid regimen, the patient was closely monitored for treatment-related adverse effects, including hyperglycemia, hypertension, electrolyte disturbances, secondary infection, gastrointestinal complications, and neuropsychiatric symptoms. Treatment was complicated by corticosteroid-induced hyperglycemia, requiring temporary insulin therapy under endocrinology supervision, while no other major corticosteroid-related complications occurred during hospitalization. The patient was discharged after 20 days of hospitalization in good general condition on a tapering regimen of oral corticosteroids. Although a substantial ulcer remained at discharge, progressive wound healing occurred during outpatient follow-up, resulting in marked tissue recovery five months after discharge. A substantial ulcer remained; however, clear wound contraction and progressive epithelialization of the entire wound were observed during follow-up (Figures 7-11). 

**Figure 7:**
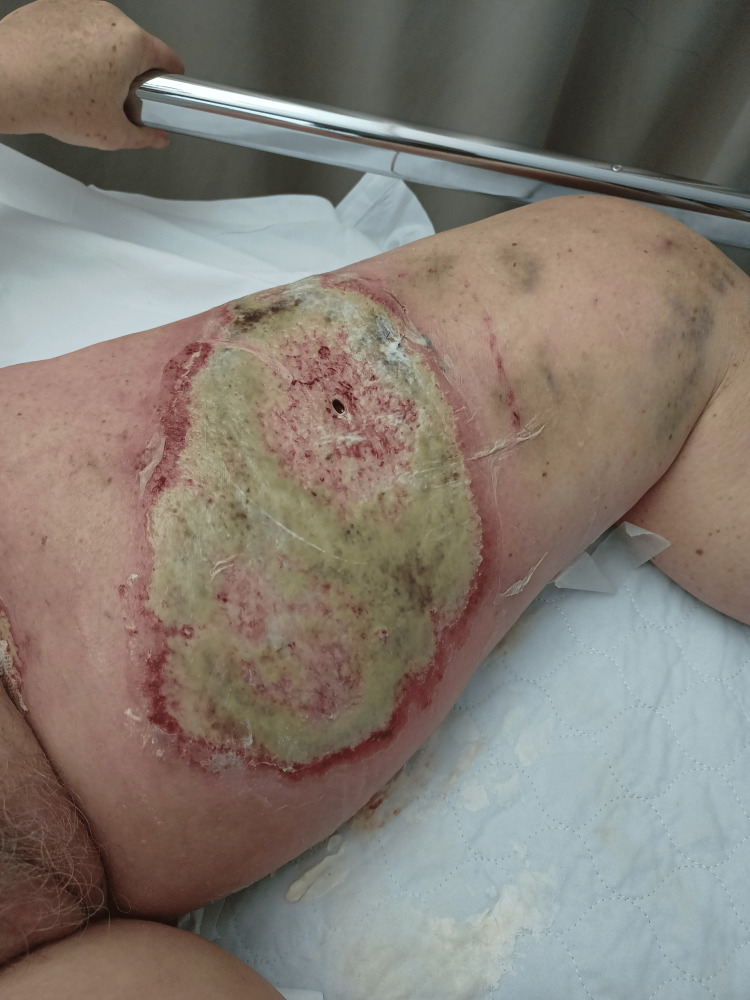
Admission day 7: immunosuppressive therapy has started - no increase of ulcer size and major decrease of inflammation, first signs of decrease of necrosis.

**Figure 8:**
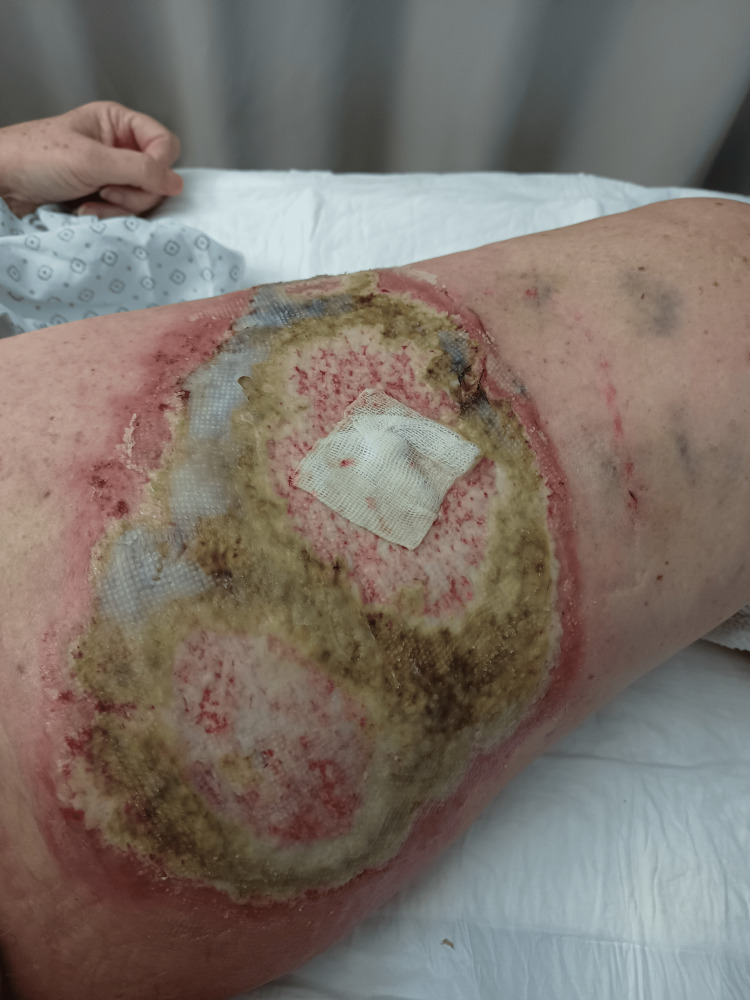
Admission day 9: after starting corticosteroids - further decrease of inflammation and necrosis, first signs of granulation.

**Figure 9:**
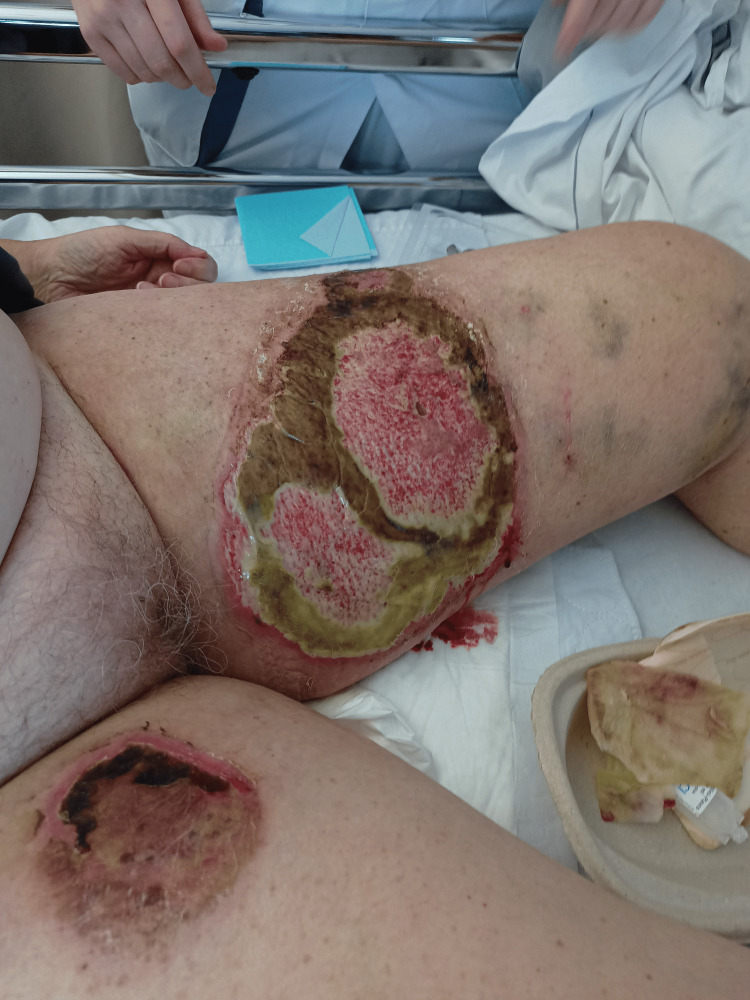
Admission day 18: further decrease of inflammation and necrosis - first signs of granulation.

**Figure 10:**
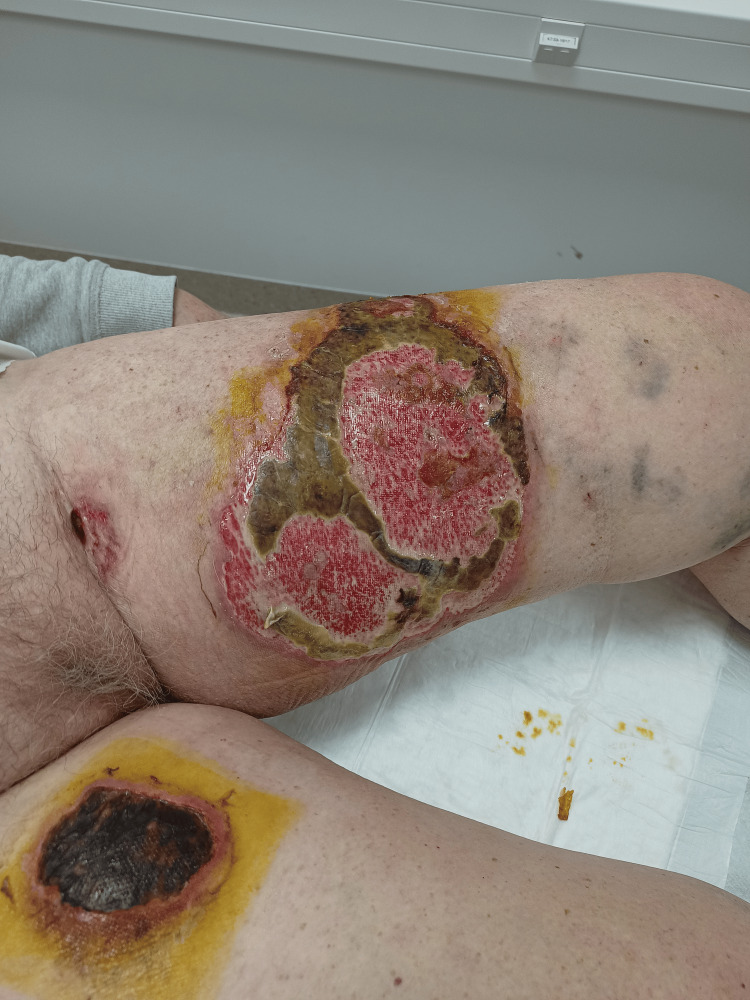
Admission day 20: tissue regeneration and granulation are also setting in from wound edges; note the extensive superficial damage without involvement of deeper tissues.

**Figure 11:**
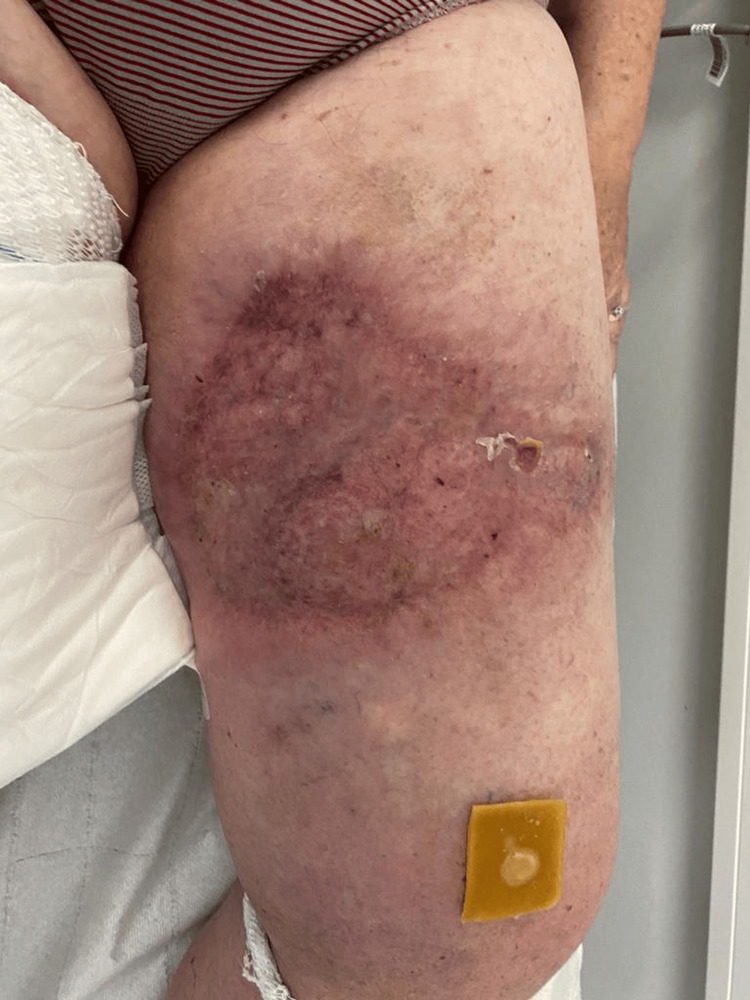
Five months after discharge: still a big wound but clear improvement - epithelialization of entire wound is visible.

In retrospect, this patient demonstrated several clinical features that should alert surgeons to the possibility of postoperative PG rather than surgical site infection, including rapidly progressive ulceration despite appropriate antimicrobial therapy, pain disproportionate to the clinical findings, persistently sterile microbiological cultures, the absence of a drainable collection on serial imaging, and a rapid clinical response following initiation of systemic corticosteroid therapy. 

## Discussion

PPG is a rare but important complication that presents a considerable diagnostic challenge for surgeons because it closely mimics postoperative wound infection. Patients commonly present with erythema, wound breakdown, purulent-appearing discharge, fever, leukocytosis, and elevated inflammatory markers, making surgical site infection the most intuitive initial diagnosis. Consequently, empirical antibiotic therapy and, when appropriate, surgical drainage or debridement are entirely appropriate during the early stages of management. However, because PPG is an autoinflammatory rather than an infectious disorder, failure to recognize the diagnosis may result in prolonged antimicrobial therapy, repeated surgical interventions, and progressive tissue destruction due to pathergy. The present case illustrates how the diagnosis evolved only after the postoperative course became inconsistent with a conventional surgical site infection [1-3].

PG is a rare neutrophilic dermatosis with an estimated incidence of three to 10 cases per million person-years. Classical PG is strongly associated with systemic disease, with a recent meta-analysis reporting an underlying comorbidity in approximately 57% of patients. The most frequent associations are inflammatory bowel disease, inflammatory arthritis, hematological malignancies, and, less commonly, solid malignancies. In contrast, PPG appears to represent a distinct clinical subtype in which surgical trauma acts as the principal trigger through the phenomenon of pathergy. Consequently, PPG is less frequently associated with systemic inflammatory disease than classic PG. In the largest published review of postoperative cases, two-thirds of patients had no identifiable underlying systemic disorder, suggesting that surgery itself may be sufficient to precipitate disease in susceptible individuals. Nevertheless, evaluation for associated systemic disease remains appropriate after the diagnosis is established, as clinically relevant comorbidities continue to occur, particularly hematological disorders. Surgical trauma is a well-recognized trigger through the phenomenon of pathergy, explaining why lesions frequently arise at incision sites and why PPG remains one of the most challenging non-infectious mimics of postoperative wound infection [2-6].

Our patient initially presented with a clinical picture that was entirely compatible with an infected postoperative hematoma or surgical site infection. Initial management therefore appropriately followed standard surgical principles, including wound drainage, microbiological sampling, local wound care, and empirical antibiotic therapy. As the disease progressed, however, several aspects of the clinical course became increasingly atypical. Despite escalation from oral to broad-spectrum intravenous antibiotics, the ulcer enlarged rapidly, inflammatory markers continued to rise, and the patient's pain became strikingly disproportionate to the local findings. Furthermore, repeated wound and blood cultures remained sterile, while serial CT examinations consistently failed to demonstrate a drainable collection, fascial gas, or other surgically treatable pathology. Although each of these findings can occur individually in other postoperative conditions, their combination suggested that an alternative diagnosis should be considered [7,8].

The diagnosis of PPG is particularly challenging because no clinical, laboratory, histopathological, or radiological finding is pathognomonic. Fever, leukocytosis, elevated inflammatory markers, and purulent-appearing wounds frequently reinforce the initial suspicion of infection, often delaying recognition of PPG. Published case series have shown that many patients undergo prolonged antibiotic treatment or repeated surgical procedures before the diagnosis is established. Consequently, the diagnosis often depends less on the initial presentation than on recognizing an unexpected clinical trajectory in which the patient fails to respond as anticipated despite appropriate treatment [8,9].

This evolving pattern represents the principal learning point of our case. Rather than relying on a single diagnostic feature, recognition of PPG depends on identifying a constellation of findings that collectively suggest an inflammatory rather than infectious process. Several clinical "red flags" have been described in the literature, including rapidly progressive ulceration despite appropriate antimicrobial therapy, severe pain disproportionate to the apparent local findings, persistently sterile microbiological cultures, and the absence of a surgically correctable focus on imaging. Our patient demonstrated each of these warning signs before the diagnosis was established. Recognition of this evolving pattern prompted multidisciplinary discussion involving dermatology and infectious disease specialists, ultimately leading to skin biopsy and initiation of immunosuppressive therapy before further tissue destruction occurred [3,8,10].

The diagnosis of PPG remains primarily clinical and is supported by histopathology together with exclusion of alternative causes. Histopathological findings are not specific and vary according to disease stage, but typically demonstrate a dense neutrophilic infiltrate while helping exclude infection, vasculitis, malignancy, and other inflammatory dermatoses. Consequently, the principal role of skin biopsy is not to establish the diagnosis independently but to support the clinical impression while excluding important differential diagnoses.

Historically, PG has been regarded as a diagnosis of exclusion, but structured diagnostic tools have improved diagnostic consistency. The Delphi consensus criteria remain the most widely accepted framework and require one major criterion - a biopsy demonstrating a neutrophilic infiltrate - together with at least four of eight minor criteria. More recently, the PARACELSUS score has been proposed as a practical diagnostic aid that incorporates rapidly progressive disease, severe pain, exclusion of relevant differential diagnoses, characteristic ulcer morphology, and response to immunosuppressive therapy. Although neither diagnostic system replaces clinical judgement, both encourage clinicians to evaluate the overall clinical picture rather than relying on individual investigations. In retrospect, our patient fulfilled the Delphi major criterion and at least five minor criteria: exclusion of infection, pathergy (development after surgery), peripheral erythema with marked tenderness, involvement of the anterior lower leg, and rapid improvement following initiation of systemic corticosteroid therapy, and achieved a PARACELSUS score of at least 14 points, well above the diagnostic threshold of 10 points. [2,7,8].

An important lesson illustrated by this case is the clinical relevance of pathergy. Minor trauma may trigger an exaggerated inflammatory response in patients with PG, resulting in rapid enlargement of existing ulcers or development of new lesions. This phenomenon has particular implications in the postoperative setting because procedures routinely performed for presumed wound infection, including debridement, wound exploration, and repeated wound manipulation, may paradoxically accelerate tissue destruction. In our patient, surgical debridement was seriously considered because of the rapidly enlarging necrotic wound. Fortunately, the increasingly atypical clinical course prompted reconsideration of the diagnosis before further surgery was undertaken. Once PPG was suspected, wound management was changed to gentle, atraumatic dressings while high-dose systemic corticosteroid therapy was initiated, after which ulcer progression rapidly ceased. Although infection should always be excluded before withholding surgery, this case demonstrates the importance of reassessing the diagnosis when the postoperative course becomes inconsistent with a conventional surgical site infection [1].

Prompt immunosuppression remains the cornerstone of treatment once infection has been reasonably excluded. Systemic corticosteroids and ciclosporin are generally considered first-line therapies for rapidly progressive or extensive disease, while biologic agents are increasingly used in refractory cases or in patients with associated inflammatory disorders. Our patient demonstrated the characteristic rapid clinical response following initiation of high-dose corticosteroids, with cessation of ulcer progression, marked improvement in pain, declining inflammatory markers, and progressive wound healing.

However, the use of high-dose systemic corticosteroids in (elderly) postoperative patients requires careful balancing of therapeutic benefit against the risks of immunosuppression. Close monitoring for steroid-related complications, including hyperglycemia and secondary bacterial infection of the open wound, is essential. In our patient, steroid-induced hyperglycemia was successfully managed with insulin therapy while vigilant surveillance for wound infection was maintained throughout treatment. This favorable response further supported the diagnosis and illustrates the importance of timely treatment once PPG is suspected [8,11,12].

Successful management extends beyond pharmacological therapy and requires close collaboration between surgeons, dermatologists, infectious disease specialists, pathologists, microbiologists, and wound care teams. In the present case, multidisciplinary discussion proved pivotal in reconsidering the diagnosis, avoiding unnecessary debridement, and initiating appropriate immunosuppressive therapy. This collaborative approach ultimately altered the patient's clinical course and limited further tissue loss [3,11,12].

This case also underscores the importance of maintaining diagnostic flexibility during postoperative care. Surgical site infection remains by far the most common cause of postoperative wound deterioration, and initial management should always prioritize exclusion and treatment of infection. Nevertheless, when patients fail to improve despite appropriate antimicrobial therapy, surgeons should be prepared to reconsider the diagnosis rather than simply escalating antibiotics or proceeding to repeated surgical intervention. Although PPG is rare, awareness of its characteristic clinical pattern may substantially shorten diagnostic delay and reduce preventable morbidity.

## Conclusions

PPG is a rare but potentially devastating complication that should be considered whenever a presumed surgical site infection follows an atypical clinical course. Progressive ulceration despite appropriate antimicrobial therapy, pain disproportionate to the clinical findings, persistently sterile microbiological cultures, and the absence of a surgically treatable focus on imaging should prompt early reconsideration of the diagnosis. Awareness of these clinical red flags, together with timely multidisciplinary collaboration and appropriate immunosuppressive therapy, may prevent unnecessary surgical intervention, limit tissue destruction, and improve patient outcomes.

Ultimately, this case highlights that PPG is not merely a rare diagnosis but a rare diagnosis that surgeons can learn to recognize earlier. Increasing awareness of this uncommon entity is essential to shorten diagnostic delay, avoid inappropriate treatment, and preserve tissue before irreversible damage occurs.
